# A hematological parameter-based model for distinguishing non-puerperal mastitis from invasive ductal carcinoma

**DOI:** 10.3389/fonc.2023.1295656

**Published:** 2023-12-13

**Authors:** Zhichun Wang, Lin Hua, Xiaofeng Liu, Xueli Chen, Guohui Xue

**Affiliations:** ^1^ Department of Breast Surgery, Jiujiang NO.1 People’s Hospital, Jiujiang, Jiangxi, China; ^2^ Department of Clinical Laboratory, Jiujiang NO.1 People’s Hospital, Jiujiang, Jiangxi, China

**Keywords:** non-puerperal mastitis, invasive ductal carcinoma, differential diagnosis, model, serological markers

## Abstract

**Purpose:**

Non-puerperal mastitis (NPM) accounts for approximately 4-5% of all benign breast lesions. Ultrasound is the preferred method for screening breast diseases; however, similarities in imaging results can make it challenging to distinguish NPM from invasive ductal carcinoma (IDC). Our objective was to identify convenient and objective hematological markers to distinguish NPM from IDC.

**Methods:**

We recruited 89 patients with NPM, 88 with IDC, and 86 with fibroadenoma (FA), and compared their laboratory data at the time of admission. LASSO regression, univariate logistic regression, and multivariate logistic regression were used to screen the parameters for construction of diagnostic models. Receiver operating characteristic curves, calibration curves, and decision curves were constructed to evaluate the accuracy of this model.

**Results:**

We found significant differences in routine laboratory data between patients with NPM and IDC, and these indicators were candidate biomarkers for distinguishing between the two diseases. Additionally, we evaluated the ability of some classic hematological markers reported in previous studies to differentiate between NPM and IDC, and the results showed that these indicators are not ideal biomarkers. Furthermore, through rigorous LASSO and logistic regression, we selected age, white blood cell count, and thrombin time to construct a differential diagnostic model that exhibited a high level of discrimination, with an area under the curve of 0.912 in the training set and with 0.851 in the validation set. Furthermore, using the same selection method, we constructed a differential diagnostic model for NPM and FA, which also demonstrated good performance with an area under the curve of 0.862 in the training set and with 0.854 in the validation set. Both of these two models achieved AUCs higher than the AUCs of models built using machine learning methods such as random forest, decision tree, and SVM in both the training and validation sets.

**Conclusion:**

Certain laboratory parameters on admission differed significantly between the NPM and IDC groups, and the constructed model was designated as a differential diagnostic marker. Our analysis showed that it has acceptable efficiency in distinguishing NPM from IDC and may be employed as an auxiliary diagnostic tool.

## 1 Introduction

Non-puerperal mastitis (NPM) is a relatively rare benign breast entity, accounting for approximately 4-5% of all benign breast lesions (1). However, the incidence and recurrence rates of NPM have rapidly increased in recent years (2, 3). NPM is a chronic inflammatory breast disease that is unrelated to pregnancy and lactation; however, its etiology remains unclear (4). Multiple factors have been associated with its occurrence, such as ductal obstruction, autoimmune system abnormalities, and infection (5–7). The course of NPM can be protracted, and some patients experience recurrence even after multiple surgical interventions (8, 9). Therefore, accurate diagnosis and timely intervention are crucial for improving the prognosis.

The clinical manifestations of NPM often present as inflammatory nodules or masses lacking the typical signs (10). During the acute phase, patients may exhibit redness, swelling, heat, pain, and fistula formation (11). Both inflammatory masses and malignant tumors can form new blood vessels, leading to a partial overlap in the clinical symptoms and imaging findings between NPM and invasive ductal carcinoma (IDC) of the breast (12, 13). Distinguishing between the two is challenging using conventional ultrasound examination. Clinical presentations of NPM during physical examination also closely resemble those of IDC of the breast, making it difficult to differentiate them based on a single indicator, thus increasing the likelihood of misdiagnosis and treatment delay. Histopathological biopsy of breast tissue is currently the only method used for a definitive diagnosis, but its acceptance rate by patients is relatively low.

Exploiting objective hematological parameters and identifying diagnostic biomarkers for differentiation are currently hot topics in the research of various diseases. This is because of the inherent advantages of easy acquisition, cost-effectiveness, and strong repeatability associated with these indicators. Research into the potential connections between these indicators and diseases may lead to the discovery of simpler and more predictive clinical parameters. Various indicators, such as neutrophil to lymphocyte ratio (NLR), platelet to lymphocyte ratio (PLR), and lymphocyte to monocyte ratio (LMR), have been found to be applicable for the auxiliary diagnosis of several diseases (14, 15). However, the discriminative capabilities of hematological indicators in distinguishing between NPM and IDC remain unclear. In recent years, the fields of regression and classification models have seen remarkable advancements, offering more accurate predictive tools. Concurrently, feature selection techniques have gained prominence for streamlining model building and reducing computational complexity (16). These methodologies are pivotal in various applications, ranging from healthcare to finance. In the realm of data science and machine learning, regression and classification models play pivotal roles in addressing a wide array of prediction and decision-making tasks. Regression models are employed for predicting continuous targets, whereas classification models are geared toward discerning discrete categories. These models span a spectrum, ranging from linear regression to deep neural networks, each excelling in distinct contexts. Feature selection methods constitute a critical phase in model construction, aiding in the identification of which features are most essential for a model’s performance (17). By eliminating redundant or irrelevant features, feature selection can enhance a model’s generalization capabilities, reduce the risk of overfitting, and expedite the training process. This study aimed to analyze the differences in hematological indicators between NPM and IDC, the discriminative abilities of previously reported serological biomarkers of inflammation, and to establish a discriminative diagnostic model based on LASSO and logistic regression or other machine learning methods.

## 2 Materials and methods

### 2.1 Subjects and study design

We conducted a retrospective review of 89 female patients between May 2012 and February 2023 who underwent biopsy or surgical procedures and were confirmed to have NPM, along with 88 female patients confirmed to have IDC and 86 female patients diagnosed with fibroadenoma (FA). Patients with incomplete clinical, laboratory, or imaging data were excluded. Additionally, patients in the NPM group were screened for other potential causes of breast inflammation, such as breast tuberculosis, fat necrosis, and inflammation due to lactation or pregnancy, and patients with these conditions were excluded from the study. All pathological characteristics of NPM, IDC, and FA were subjected to a double-blind random review by two pathologists. The NPM cases were diagnosed in accordance with the Chinese Society of Breast Surgery (CSBrS) 2021 practice guidelines (8). This study was approved by the Ethics Committee of Jiujiang No.1 People’s Hospital. Considering the non-interventional retrospective nature of this study and the utilization of electronic record data coupled with the anonymization of patient information, informed consent from the patients involved was not required.

### 2.2 Laboratory data collection

We collected blood biochemical, hematological, and coagulation indicators as well as the age of all study subjects upon admission for treatment. Blood biochemical tests encompassed alanine aminotransferase (ALT), aspartate aminotransferase (AST), alkaline phosphatase (ALP), γ-glutamyltransferase (GGT), total protein (TP, g/L), albumin (ALB), prealbumin (PA), globulin (GLOB), total bilirubin (TBIL), direct bilirubin (DBIL), indirect bilirubin (IBIL), glucose (GLU), urea, creatinine (CREA), uric acid (URCA), creatine kinase (CK), creatine kinase-MB (CKMB), lactate dehydrogenase (LDH), and α-hydroxybutyrate dehydrogenase (HBDH), which were measured using a Hitachi 7600 automated biochemical analyzer (Hitachi Limited Co., Japan). Hematological tests included white blood cell count (WBC), red blood cell count (RBC), hemoglobin (Hb), hematocrit (Hct), mean corpuscular volume (MCV), mean corpuscular hemoglobin (MCH), mean corpuscular hemoglobin concentration (MCHC), red cell distribution width coefficient of variation (RDW-CV), platelet count (PLT), mean platelet volume (MPV), percentage of neutrophils (NEU%), percentage of lymphocytes (LYM%), percentage of monocytes (MON%), neutrophil count (NEU), lymphocyte count (LYM), monocyte count (MON), eosinophil count (EOS), and basophil count (BAS), which were determined using a Sysmex XN-2000 automated hematology analyzer (Sysmex Limited Co., Japan). Coagulation function indicators, including prothrombin time (PT), activated partial thromboplastin time (APTT), thrombin time (TT), and fibrinogen (Fbg) were determined using a CS-5100 automated blood coagulation analyzer (Sysmex Limited Co., Japan). All laboratory indicators were measured in accordance with standard operating procedures in the clinical laboratory of our hospital, and these indicators are listed in **Table 1**.

**Table 1:** The biomedical indicators, routine blood parameters and coagulation indicators of NPM, IDC and FA patients.

Indexes	NPM (n=89)	IDC (n=88)	FA (n=86)	p1,p2,p3
Age (years)	35.96 ± 9.52	52.55 ± 12.75	39.36 ± 11.02	<0.0001, <0.05, <0.0001
ALT (U/L)	18.68 ± 17.97	20.88 ± 17.55	17.52 ± 10.92	ns, ns, ns
AST (U/L)	17.81 ± 6.40	21.75 ± 8.52	19.43 ± 6.09	<0.0001, <0.05, ns
ALP (U/L)	76.45 ± 26.16	70.38 ± 22.34	59.97 ± 16.78	ns, <0.001, <0.01
GGT (U/L)	21.8 ± 15.33	29.40 ± 48.67	20.15 ± 13.92	ns, ns, ns
TP (g/L)	73.15 ± 4.70	73.84 ± 3.95	74.56 ± 3.70	ns, ns, ns
ALB (g/L)	44.74 ± 4.18	45.49 ± 2.46	45.83 ± 2.30	ns, ns, ns
PA (mg/L)	229.63 ± 57.10	265.24 ± 48.84	264.03 ± 38.57	<0.0001, <0.0001, ns
GLOB (g/L)	28.09 ± 4.12	28.35 ± 3.20	28.73 ± 3.07	ns, ns, ns
TBIL (μmol/L)	9.98 ± 4.35	13.04 ± 6.26	12.23 ± 4.19	<0.001, <0.001, ns
DBIL (μmol/L)	3.22 ± 1.46	3.49 ± 1.75	3.44 ± 1.23	ns, ns, ns
IBIL (μmol/L)	6.70 ± 3.11	9.56 ± 4.64	8.79 ± 3.11	<0.0001, <0.0001, ns
GLU (mmol/L)	5.17 ± 0.65	5.74 ± 1.66	5.18 ± 0.87	<0.05, ns, <0.01
Urea (mmol/L)	4.09 ± 1.21	5.11 ± 1.73	4.48 ± 1.20	<0.0001, ns, <0.05
CREA (μmol/L)	52.01 ± 8.86	57.25 ± 21.01	53.01 ± 7.04	<0.05, ns, ns
URCA (μmol/L)	284.97 ± 72.56	272.08 ± 62.59	276.05 ± 65.93	ns, ns, ns
CK (U/L)	65.55 ± 27.76	82.50 ± 32.7	73.49 ± 27.24	<0.01, ns, ns
CKMB (U/L)	11.65 ± 5.57	11.86 ± 4.12	10.21 ± 2.73	ns, ns, <0.01
LDH (U/L)	169.93 ± 32.47	181.28 ± 36.58	166.92 ± 26.52	<0.05, ns, <0.01
HBDH (U/L)	140.38 ± 27.21	146.89 ± 38.02	135.97 ± 24.73	ns, ns, <0.01
WBC (10^9^/L)	7.78 ± 3.13	5.52 ± 1.40	5.65 ± 1.44	<0.0001, <0.0001, ns
RBC (10^12^/L)	4.38 ± 0.49	4.45 ± 0.40	4.48 ± 0.37	ns, ns, ns
Hb (g/L)	124.83 ± 14.54	129.68 ± 16.84	135.33 ± 12.48	<0.05, <0.0001, ns
Hct (%)	0.39 ± 0.04	0.40 ± 0.04	0.40 ± 0.03	ns, <0.05, ns
MCV (fL)	87.75 ± 6.34	89.02 ± 7.11	90.01 ± 5.1	ns, <0.05, ns
MCH (pg)	28.28 ± 2.79	29.28 ± 3.32	30.26 ± 2.45	<0.01, <0.0001, ns
MCHC (g/L)	320.3 ± 15.46	326.82 ± 18.87	336.09 ± 14.36	<0.01, <0.0001, <0.05
RDW-CV (%)	13.26 ± 1.74	13.16 ± 1.73	12.84 ± 1.50	ns, ns, ns
PLT (10^9^/L)	260.17 ± 63.38	238.47 ± 73.32	244.69 ± 73.05	<0.05, ns, ns
MPV (fL)	10.91 ± 1.28	10.84 ± 0.95	10.9 ± 1.03	ns, ns, ns
PDW	13.89 ± 2.73	12.98 ± 2.25	13.15 ± 2.30	<0.05, <0.05, ns
NEU%	67.57 ± 9.46	62.90 ± 7.66	60.74 ± 7.89	<0.001, <0.0001, ns
LYM%	24.26 ± 8.40	27.76 ± 7.04	30.52 ± 7.57	<0.01, <0.0001, ns
MON%	6.24 ± 1.73	6.67 ± 1.84	6.58 ± 1.57	ns, ns, ns
NEU (10^9^/L)	5.43 ± 2.88	3.53 ± 1.22	3.60 ± 1.55	<0.0001, <0.0001, ns
LYM (10^9^/L)	1.70 ± 0.50	1.49 ± 0.40	1.69 ± 0.49	<0.01, ns, <0.05
MON (10^9^/L)	0.48 ± 0.23	0.36 ± 0.13	0.36 ± 0.10	<0.001, <0.01, ns
EOS (10^9^/L)	0.1 ± 0.07	0.08 ± 0.08	0.09 ± 0.10	<0.01, <0.05, ns
BAS (10^9^/L)	0.03 ± 0.02	0.03 ± 0.02	0.03 ± 0.02	ns, ns, ns
PT (s)	11.34 ± 1.24	11.35 ± 0.78	11.31 ± 0.76	ns, ns, ns
APTT (s)	27.07 ± 4.14	26.20 ± 2.36	27.64 ± 2.71	ns, ns, <0.01
Fbg (g/L)	3.23 ± 0.92	2.67 ± 0.70	2.65 ± 0.61	<0.001, <0.0001, ns
TT (s)	18.44 ± 3.70	17.07 ± 1.33	17.85 ± 2.41	<0.05, ns, ns

p1, NPM vs IDC; p2, NPM vs FA; p3, IDC vs FA; ns, no significant difference.

### 2.3 Derived serological markers of inflammation

We have compiled a summary of previously reported hematological inflammatory biomarkers in the literature (14, 18, 19), such as the NLR (NEU counts/LYM counts), PLR (PLT counts/LYM counts), LMR (LYM counts/MON counts), derived neutrophil-lymphocyte ratio (dNLR, NEU counts/(WBC counts-NEU counts)), albumin-to-fibrinogen ratio (AFR, ALB/Fbg), prognostic nutritional index (PNI, 10 × ALB + 5 × LYM counts), systemic immune-inflammation index (SII, PLT counts × NEU counts/LYM counts), aggregate index of systemic inflammation (AISI, NEU counts × MON counts × PLT counts/LYM counts), neutrophil-to-lymphocyte platelet ratio (NLPR, NEU counts/LYM counts × PLT counts), systemic inflammation response index (SIRI, NEU counts × MON counts/LYM counts), neutrophil-monocyte ratio (NMR, NEU counts/MON counts), and neutrophil-to-eosinophil ratio (NER, NEU counts/EOS counts), among others. We analyzed the performance of these biomarkers in distinguishing NPM from IDC or FA.

### 2.4 Statistical analysis

The statistical software SPSS 23.0 and GraphPad Prism 8.0.2 were utilized for all data analysis. The independent samples t-test was applied to analyze differences in continuous variables between the two groups if the data met a normal distribution; otherwise, the Mann-Whitney U test was performed. LASSO and logistic regression were used to screen the parameters for the construction of the diagnostic model. To assess the model’s capability, the receiver operating characteristic (ROC), calibration, and decision curves were plotted using R Project 4.0.2. A two-tailed *p* value of < 0.05 was considered statistically significant. Machine learning methods such as random forest, decision tree, and SVM were also utilized to construct discriminative diagnostic models.

## 3 Results

### 3.1 Comparison of routine laboratory data for the study populations

In line with prior epidemiological investigations, patients with IDC exhibited a notably higher average age compared with patients with NPM. Additionally, the average age of the patients in the FA group was notably higher than that in the NPM group. Blood biochemistry tests revealed that patients with NPM exhibited lower levels of AST, TBIL, IBIL, GLU, Urea, CREA, CK, and LDH than patients with IDC. Regarding routine blood parameters, patients with NPM exhibited higher levels of WBC, PLT, PDW NEU%, NEU, LYM, MON, and EOS, along with lower levels of Hb, MCH, MCHC, and LYM% compared to patients with IDC. When comparing the coagulation indices between the two groups, patients with NPM exhibited higher levels of Fbg and TT. Likewise, NPM patients exhibited significant differences in certain indicators compared to FA patients, as shown in **Table 1**.

### 3.2 Performance evaluation of derived serological markers for differential diagnosis

**Figure 1:**
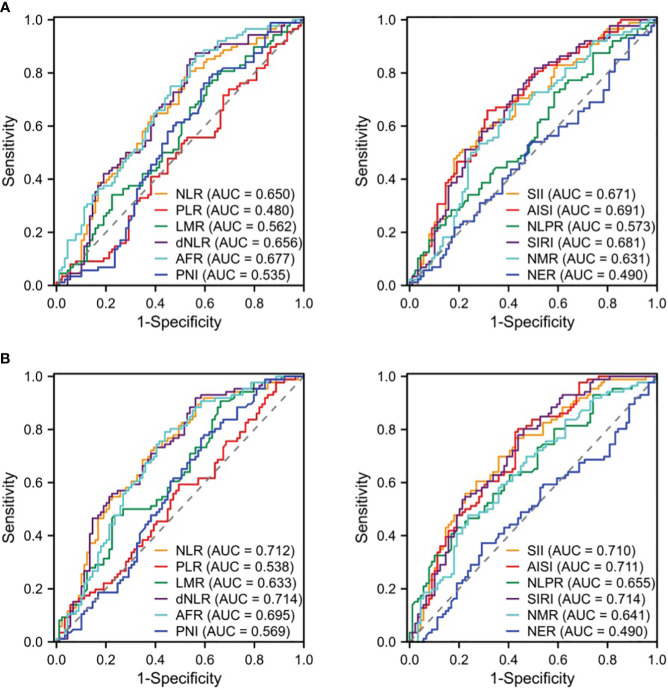
ROC curves of derived serologic markers for identification of NPM vs. IDC **(A)** or FA **(B)**. NLR, neutrophil to lymphocyte ratio; PLR, platelet to lymphocyte ratio; LMR, lymphocyte to monocyte ratio; dNLR, derived neutrophil-lymphocyte ratio; AFR, albumin-to-fibrinogen ratio; PNI, prognostic nutritional index; SII, systemic immune-inflammation index; AISI, aggregate index of systemic inflammation; NLPR, neutrophil-to-lymphocyte platelet ratio; SIRI, systemic inflammation response index; NMR, neutrophil-monocyte ratio; NER, neutrophil-to-eosinophil ratio; ROC, receiver operating characteristic curves; AUC, area under ROC curve.

We compared the differences in the derived serological markers among the three groups of patients and found that, compared to the IDC and FA groups, patients with NPM exhibited higher levels of NLR, dNLR, SII, AISI, SIRI, and NMR, along with lower levels of AFR (**Table 2**). ROC curves indicated that, for distinguishing between NPM and IDC, the AUC^ROC^ values for these derived serological markers were all below 0.70, suggesting poor diagnostic performance (**Figure 1A**). When distinguishing NPM from FA, although the NLR, dNLR, SII, AISI, and SIRI were all above 0.70, they remained below 0.72, indicating a moderate diagnostic performance (**Figure 1B**). Therefore, it is necessary to explore new biomarkers or models with better discriminatory capability.

**Table 2:** The derived serological markers in NPM, IDC, and FA patients.

Indexes	NPM (n=89)	IDC (n=88)	FA (n=86)	p1,p2,p3
NLR	3.4 ± 2.1	2.53 ± 1.21	2.29 ± 1.32	<0.01, <0.0001, ns
PLR	166.66 ± 72.36	169.92 ± 73.13	152.5 ± 48.49	ns, ns, ns
LMR	4.1 ± 1.69	4.45 ± 1.67	4.91 ± 1.75	ns, <0.01, ns
dNLR	2.37 ± 1.2	1.84 ± 0.76	1.63 ± 0.72	<0.01, <0.0001, ns
AFR	15.14 ± 5.02	18.23 ± 5.01	18.17 ± 4.21	<0.001, <0.0001, ns
PNI	455.96 ± 42.13	462.4 ± 24.97	466.74 ± 23.65	ns, ns, ns
SII	926.07 ± 707.41	607.07 ± 340.77	554.36 ± 322.7	<0.001, <0.0001, ns
AISI	520.84 ± 647.05	233.92 ± 179.35	205.52 ± 126.28	<0.0001, <0.0001, ns
NLPR	0.01 ± 0.01	0.01 ± 0.01	0.01 ± 0.01	ns, ns, ns
SIRI	1.86 ± 2.08	0.97 ± 0.76	0.83 ± 0.46	<0.001, <0.0001, ns
NMR	11.69 ± 3.95	10.08 ± 3.08	10.45 ± 6.09	<0.05, <0.01, ns
NER	93.66 ± 122.28	87.18 ± 115.11	78.35 ± 71.13	ns, ns, ns

p1, NPM vs IDC; p2, NPM vs FA; p3, IDC vs FA; ns, no significant difference. NLR, neutrophil to lymphocyte ratio; PLR, platelet to lymphocyte ratio; LMR, lymphocyte to monocyte ratio; dNLR, derived neutrophil-lymphocyte ratio; AFR, albumin-to-fibrinogen ratio; PNI, prognostic nutritional index; SII, systemic immune-inflammation index; AISI, aggregate index of systemic inflammation; NLPR, neutrophil-to-lymphocyte platelet ratio; SIRI, systemic inflammation response index; NMR, neutrophil-monocyte ratio; NER, neutrophil-to-eosinophil ratio.

### 3.3 Screening of key indicators and construction of models for differential diagnosis

**Figure 2:**
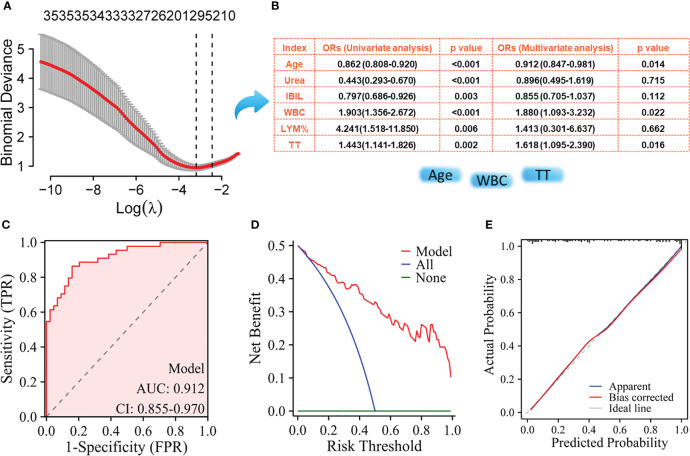
Screening of key indicators for building the model to distinguish NPM from IDC and its performance evaluation using the training set. **(A)** LASSO logistic regression model; **(B)** Results of univariate logistic regression and multivariate logistic regression; **(C)** ROC curves; **(D)** Decision curve analysis; **(E)** Calibration curves.

**Figure 3:**
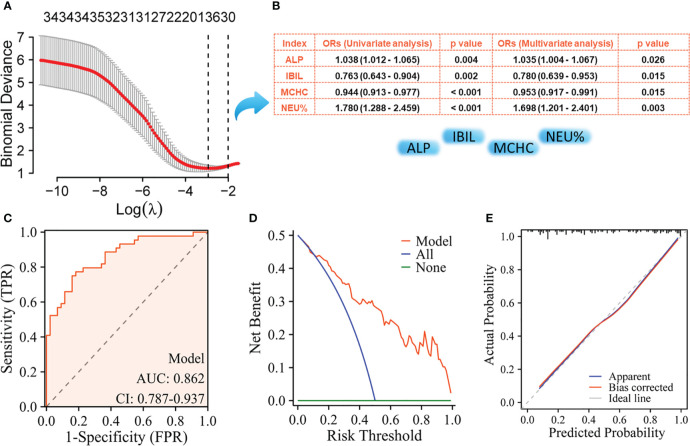
Screening of key indicators for building the model to distinguish NPM from FA and its performance evaluation using the training set. **(A)** LASSO logistic regression model; **(B)** Results of univariate logistic regression and multivariate logistic regression; **(C)** ROC curves; **(D)** Decision curve analysis; **(E)** Calibration curves.

The NPM patients and IDC patients were randomly divided into a training set (NMP, n= 44; IDC, n= 44) and a validation set (NMP, n= 45; IDC, n= 44) in a 1:1 ratio, where the training set was used to build the model, and the validation set was used to verify the accuracy of the model. Using LASSO regression to select key variables for model construction, with the principle of keeping the model concise under lambda compression (lambda.1se), variables with small regression coefficients were directly compressed to zero to eliminate corresponding variables (**Figure 1A**). To construct models to distinguish NPM from IDC, the key variables used were age, IBIL,Urea, WBC count, LYM%, and TT (**Figure 1B**). Subsequently, univariate and multivariate logistic regression were performed on these indicators, the parameters of age, WBC, and TT were ultimately selected for model construction. The ROC curve showed an AUC of 0.912 (**Figure 2C**), and both the calibration (**Figure 2D**) and decision curves (**Figure 2E**) demonstrated that this model was reliable for distinguishing between NPM and IDC. Furthermore, the NPM patients and FA patients were randomly divided into a training set (NMP, n= 44; FA, n= 43) and a validation set (NMP, n= 45; FA, n= 43) in a 1:1 ratio, and using the same key parameter selection method and model construction approach, a model for distinguishing between NPM and FA was constructed (**Figure 3A and B**). The ROC curve showed an AUC of 0.862 (**Figure 3C**), and the calibration (**Figure 3D**) and decision curves (**Figure 3E**) indicated good performance in distinguishing between NPM and FA.

**Figure 4:**
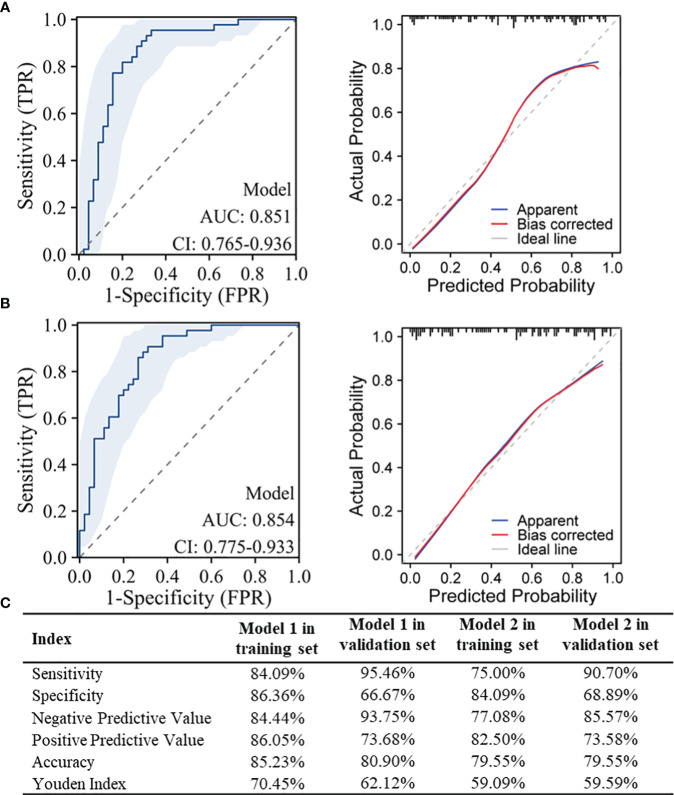
Performance testing of the models in the validation set and diagnostic performance parameters in the training and validation sets. **(A)** ROC curves and Calibration curves for model 1 used to distinguish between NPM and IDC in the validation set; **(B)** ROC curves and Calibration curves for model 2 used to distinguish between NPM and FA in the validation set; **(C)** Diagnostic performance parameters for model 1 and model 2 in the training and validation sets.

**Figures 4A, B** listed the performance parameters of the two diagnostic models in the validation set, and the diagnostic performance parameters for model 1 and model 2 in the training and validation sets were shown in **Figure 4C**. For model 1 used to distinguish between NPM and IDC, it also demonstrated a high AUC of 0.851 in the validation set, while model 2 used to distinguish between NPM and FA achieved an even higher AUC of 0.854 in the validation set.

### 3.4 Using machine learning approaches to construct the diagnostic discrimination models

**Figure 5:**
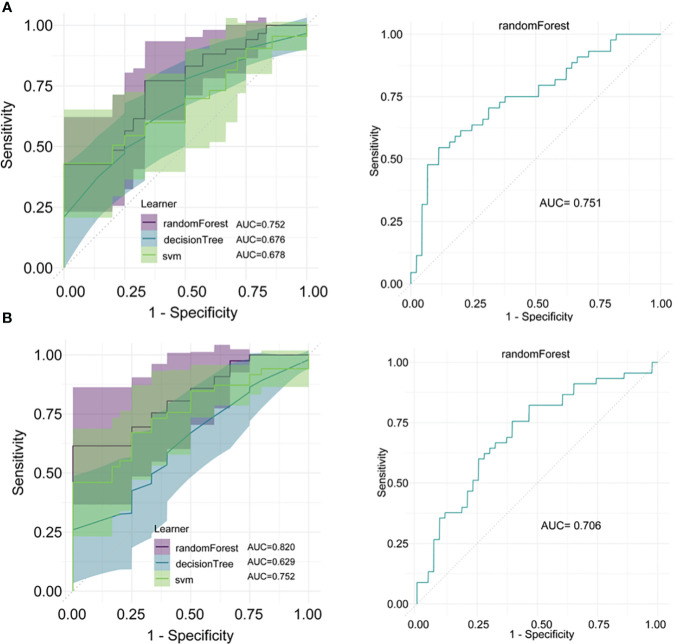
Model construction and validation using machine learning methods. **(A)** Models for distinguishing between NPM and IDC established using random forest, decision tree, and SVM, and the ROC curve for the random forest model in the validation set; **(B)** Models for distinguishing between NPM and FA established using random forest, decision tree, and SVM, and the ROC curve for the random forest model in the validation set.

Using three machine learning methods, random forest, decision tree, and SVM, models were constructed in the training sets. The results showed that for distinguishing between NPM and IDC, the random forest model achieved the highest AUC in the training set at 0.752 and in the validation set at 0.751 (**Figure 5A**). For distinguishing between NPM and FA, in the training set, the random forest model achieved the highest AUC at 0.820, and in the validation set, it was 0.706 (**Figure 5B**). In both the training and validation sets, these three machine learning models performed lower than the models established in this study using lasso and logistic regression.

## 4 Discussion

NPM is a benign, non-tumorous, non-specific inflammatory breast condition characterized by ductal dilation, extensive infiltration of inflammatory cells, and late-stage ductal and adjacent tissue infiltration and proliferation (20, 21). NPM primarily affects non-lactating women aged between 30 and 40 (22). In clinical practice, NPM is relatively uncommon but has been on the rise in recent years. Clinically, only some patients exhibit symptoms such as redness, swelling, and pain, whereas most present with breast lumps accompanied by pain and lack the typical clinical features (23). IDC, the most common type of breast cancer, typically manifests as a hard lump with unclear borders, poor mobility, and some degree of pain. Consequently, there is an overlap in the clinical presentations of NPM and IDC (24, 25). Owing to its real-time and noninvasive nature, ultrasound has become an important tool for routine breast examination in women (26, 27). However, two-dimensional and color Doppler ultrasound images of both NPM and IDC can show hypoechoic or mixed-echo masses, indistinct borders, irregular shapes, uneven internal echoes, and ductal dilation, leading to imaging overlap (28). Interpretation of these imaging features can also be influenced by the physician’s subjective experience. Therefore, reliance on ultrasound can make differential diagnosis challenging. Research has shown that NPM is frequently confused with IDC with a high preoperative misdiagnosis rate. Importantly, the treatment approaches for these conditions are markedly different. Therefore, the ability to accurately differentiate NPM from IDC preoperatively is of crucial clinical significance for the diagnosis and treatment of patients with NPM. Numerous studies have demonstrated the diagnostic value of serologically derived biomarkers for various diseases, including cancer. However, it is currently unclear whether hematological markers can serve as discriminative biomarkers to distinguish NPM from IDC.

NLR, PLR, LMR, dNLR, AFR, PNI, SII, AISI, NLPR, SIRI, NMR, NER, and other serological markers have previously demonstrated utility in the differential diagnosis and prognostic assessment of various cancers, including breast cancer (29–31). However, whether these parameters can differentiate between NPM and IDC remains unclear. Based on the ROC curve analysis, we found that these markers had relatively poor discriminative performance and only exhibited moderate performance when distinguishing NPM from FA. This suggests an urgent need to identify new blood parameters with better discriminative diagnostic capabilities. Initial laboratory indicators upon admission and before treatment can, to some extent, reflect the true condition of different diseases. In this study, we assessed differences in laboratory characteristics between patients with NPM and those with IDC at the time of admission. Through statistical analysis, we identified several markers that showed significant differences between groups. These markers can serve as important auxiliary references for differential diagnosis, especially when there is an overlap in imaging or clinical symptoms. However, there are more than 20 differentiating markers, which may not be practical for clinical applications. Using LASSO and logistic regression, we successfully identified the most crucial components for distinguishing between these two diseases: age, WBC count, and TT. Using these three indicators, we successfully constructed a differential diagnostic model, and the ROC curve confirmed an AUC of 0.912, with a sensitivity of 84.09% and a specificity of 86.36%. Our model outperformed the other models. For instance, Tang et al. (12) used the Magnetic Resonance Imaging (MRI) volumetric apparent diffusion coefficient to differentiate between NPM and breast cancer with an AUC of only 0.821, which was lower than the AUC of our model. Similarly, another model using grayscale ultrasound (GSUS) and contrast-enhanced ultrasound (CEUS) images achieved an AUC of approximately 0.80 (32), which was also lower than our study’s model, and their model’s performance significantly improved when clinical parameters such as age and NEU were incorporated. Our model also included age as a critical factor. Both univariate and multivariate analyses indicated that NPM was more common in younger women, whereas IDC was more prevalent in older women. These findings are consistent with those of previous studies (32, 33).

Although the exact etiology of NPM remains unclear, it is a benign inflammatory disease different from IDC (20). Therefore, blood cell indices can serve as indicators of systemic inflammation and can differentiate between NPM and IDC. Our data showed that NPM patients had significantly higher WBC counts than IDC patients. The WBC count is a non-specific marker of inflammation and can indicate active bacterial infection. Patients with IDC rarely present with active bacterial infections. Thus, WBC count can be used to distinguish NPM from IDC.

This study has certain limitations. First, it was a single-center retrospective study with a relatively narrow cohort size owing to the low incidence rate of NPM, and large-scale studies and multicenter research are needed to thoroughly validate the reliability and clinical value of our model in the future. Second, the laboratory indicators included were not sufficiently comprehensive, as some were missing from the IDC cases. Nonetheless, based on these simple indicators, a model with acceptable efficiency was constructed.

In conclusion, we successfully developed a model that can effectively differentiate NPM from IDC. For the clinical differential diagnosis of NPM, this model can serve as an effective adjunct to imaging examinations and may help avoid unnecessary biopsies. Furthermore, incorporating this model may improve the current laboratory diagnostic criteria recommended by the NPM guidelines.

## Data availability statement

The raw data supporting the conclusions of this article will be made available by the authors, without undue reservation.

## Ethics statement

The studies involving humans were approved by the ethics committee of Jiujiang NO.1 People’s Hospital. The studies were conducted in accordance with the local legislation and institutional requirements. Written informed consent for participation was not required from the participants or the participants’ legal guardians/next of kin in accordance with the national legislation and institutional requirements.

## Author contributions

GX: Conceptualization, Funding acquisition, Software, Supervision, Validation, Visualization, Writing – review & editing. ZW: Investigation, Methodology, Project administration, Resources, Writing – original draft. LH: Data curation, Investigation, Methodology, Project administration, Software, Visualization, Writing – original draft. XL: Data curation, Software, Validation, Visualization, Writing – review & editing. XC: Data curation, Investigation, Writing – review & editing.
